# The Prevalence of Left-Handedness Is Higher Among Individuals With Developmental Coordination Disorder Than in the General Population

**DOI:** 10.3389/fpsyg.2018.01948

**Published:** 2018-10-18

**Authors:** Monica Darvik, Håvard Lorås, Arve Vorland Pedersen

**Affiliations:** Department of Neuromedicine and Movement Science, Faculty of Medicine and Health Sciences, Norwegian University of Science and Technology, Trondheim, Norway

**Keywords:** laterality, dextrality, sinistrality, clumsiness, dyspraxia, hand dominance, preference

## Abstract

Many medical, psychiatric and neurological conditions have been characterized by a high prevalence of left-handedness or mixed-handedness. Several studies have indicated an elevated frequency of left-handedness in children with Developmental Coordination Disorder (DCD). However, there have been few studies explicitly exploring this relationship. The assumption is that the prevalence of left-handedness in individuals with DCD is higher compared with the prevalence in the general population and resembles the prevalence described in children with other developmental disorders. Computerized searches were conducted in PubMed, PsycInfo and CINAHL databases. Thirty-eight studies were identified and included in the present review, containing handedness distributions across 1071 persons with DCD and 1,045 controls. The distribution of DCD participants across handedness-categories was proved to be significantly different from that of the control group, with 14.7 and 8.1% left-handers, respectively. The prevalence of left-handedness within the DCD-group is lower than that reported for ASD, and larger than in dyslexia. The elevated levels of left-handedness within the different developmental disorders supports the notion of an association between the different diagnoses. However, the present results are not sufficient to conclude anything about a common cause or underlying factor via the male hormone testosterone. The present results could act as a starting point for testing the hypothesis of such a common factor, as one of the requirements is an elevated prevalence of left-handedness, and without such considerable doubt would be cast upon the hypothesis.

## Introduction

Among developmental disorders, Developmental Coordination Disorder (DCD) is less understood (Gomez and Sirigu, 2015). DCD is a neurodevelopmental motor disorder. Symptoms typically onset in the early developmental period, with motor skills substantially below expectations given the individual's age. The deficits in motor skills impacts activities of daily living and are not attributable to a neurological condition affecting movement, nor can they be explained by visual impairment or intellectual disability (American Psychiatric Association, 2013). DCD is usually a permanent condition found in children, affecting between 5 and 8% between 6 and 12 years of age (Barnhart et al., 2003; Noten et al., 2014). A higher prevalence is found in boys than girls (Kadesjö and Gillberg, 1999; Barnhart et al., 2003). DCD is also more common in low-birth-weight children and those with prenatal exposure to alcohol (American Psychiatric Association, 2013).

DCD has a developmental rather than acquired origin, with difficulty in coordination and control of voluntary motor activity in the absence of intellectual impairment and neurological and/or physical disorder (Cermak et al., 2002; Gibbs et al., 2007). Other terms previously used to describe DCD include clumsy child syndrome, childhood dyspraxia and specific developmental disorder of motor function (American Psychiatric Association, 2013).

DCD is often associated with psychopathology (Gillberg and Kadesjö, 2003; Goez and Zelnik, 2008). A shared genetic effect has been proposed because of the co-occurrence of DCD with autism spectrum disorder, specific learning disabilities and attention-deficit/hyperactivity disorder (ADHD). However, such consistency in co-occurrence in twins appears only in severe cases (American Psychiatric Association, 2013). Several studies have shown a rate of about half of the children with ADHD also having DCD, and there is a growing idea that DCD may not be a uniform disorder (see Visser, 2003). An alternative view regarding the classification of developmental disorders, such as ASD, ADHD, and DCD, is that there is one group of children with heterogeneous, atypical brain development, rather than discrete groups of children (Gillberg and Kadesjö, 2003; Goez and Zelnik, 2008; Vaivre-Douret et al., 2016). An association between DCD and ADHD (Denckla, 1996) and motor control has been reported. Motor control problems may also be part of ASD (Gillberg and Kadesjö, 2003; Whyatt and Craig, 2013). The symptoms of the respective diseases also overlap, which is reflected in the earlier term Minimal Brain Dysfunction (MDB), labeling syndromes with various combinations of deficits in motor control, language, memory, perception, memory and impulse control (Gillberg and Kadesjö, 2003). According to the DSM V, the motor skills deficits should not be better explained by intellectual disability (intellectual developmental disorder) or visual impairment, and they should not be attributable to a neurological condition affecting movement (American Psychiatric Association, 2013). Still, neurological deficits have been reported in children with DCD, including slowed muscle force production and sensory organization deficits (Fong et al., 2015, see also Adams et al., 2014 for a review on internal modeling deficits). Furthermore, these children may demonstrate symptoms of neurological “soft signs” (Dewey, 2002). Such signs reflect minor neurological abnormalities and include dysdiadochokinesia, synkinesia, tactile localization deficits, reduced motor speed, mild dysfunction in muscle tone regulation, and asymmetric reflexes (Shaffer et al., 1985; Vaivre-Douret et al., 2016).

According to Geschwind and Behan (1982), there is an increased prevalence of left-handedness in patients with immune diseases, migraine and learning difficulties due to delayed growth in the left hemisphere caused by testosterone, which interferes with language functions and creates a shift of handedness toward the right hemisphere. Testosterone also accounts for the much greater prevalence of learning disabilities in boys. Also, according to Llaurens et al. (2009), one underlying causal factor of left-handedness may be low birth weight, which is associated with perinatal difficulties. Prenatal factors have also been proposed to cause left-handedness (Geschwind and Behan, 1982; Llaurens et al., 2009; Parma et al., 2017). Children with extreme low birth weights and children born prematurely have a significantly increased risk of demonstrating DCD (Barnhart et al., 2003; Gibbs et al., 2007; Kwok et al., 2018).

Handedness is regarded as one of the most evident lateral behavioral traits (Triggs et al., 2000). Lateralization denominates the processes that lead to an asymmetrical nervous system (Geschwind and Galaburda, 1985), with the end-product often being referred to as laterality. Handedness, which can be defined as “the individual's preference to use one hand predominantly for unimanual tasks and the ability to perform these tasks more efficiently with one hand” (Brown et al., 2006, p.1). Hand preference can be defined as a greater preference of one hand over the other if a choice is possible (Peters, 1995). Roughly 90% of the healthy adult population prefers using their right hand for manual actions (Cavill and Bryden, 2003; Adamo and Taufiq, 2011; Ooki, 2014; Scharoun and Bryden, 2014; Willems et al., 2014). However, inconsistent handedness is more prevalent among left-handers than right-handers, and males also tend to be more inconsistently handed than females (Prichard et al., 2013).

Several studies have indicated an elevated frequency of left-handed DCD children, and left-handedness, crossed dominance, mixed preference and poorly established hand preferences have all been linked with clumsiness (Armitage and Larkin, 1993). However, there are few studies that explicitly explore such relationships. Hill and Bishop (1998) stated that handedness had not been investigated directly in the DCD population. In their study, groups of children with DCD and specific language impairment (SLI) did not differ in terms of hand preference. Ten years later, Cairney et al. (2008) also concluded that “we know of no published work that has used objective clinical assessments of both handedness and DCD” (p. 697). However, Goez and Zelnik (2008) investigated the distribution of hand dominance in 98 children with DCD. They concluded that children with DCD are more often left-handed compared with the general population. A more recent study investigated handedness and DCD in Portuguese children (Freitas et al., 2014). These authors reported a higher co-occurrence rate of left-handedness compared to right-handedness in DCD children. It should, however, be noted that Freitas et al. (2014) deliberately recruited left-handers. Nevertheless, there are also studies concluding there is no higher prevalence of left-handedness in clumsy children (e.g., Armitage and Larkin, 1993).

Freitas et al. (2014) emphasized that the “literature's general tendency suggests an association between DCD and left-handed children, but it still remains unclear” (p. 657). Handedness found in other comorbid diagnoses may give an indication of what to expect in DCD. In a review of 12 studies including a total of 497 individuals diagnosed with ASD, Rysstad and Pedersen (2016) found 16% left-handers and 44% mixed-handers, giving a total of 60% non-right-handers. Furthermore, in a meta-analysis of dyslexia, Eglinton and Annett (1994) reported a significant difference in handedness distribution between dyslectics and control groups. The proportion of left-handers was approximately equal in both groups, with 10.7 and 10.4% left-handers. However, there were more than twice as many dyslexics (11.7%) with mixed-handedness than in the control groups (5.4%), which means that the difference in mixed-handers alone accounts for the distribution differences between the groups (Eglinton and Annett, 1994). In ADHD, in contrast, the findings about the association between left-handedness and disease are inconsistent (Ghanizadeh, 2013). Left-handedness has been proposed as a risk-factor for ADHD and reported as markedly preferred (Niederhofer, 2005), while others have failed to confirm this association (Biederman et al., 1995; Ghanizadeh, 2013).

Given the association between left- and mixed-handedness and other developmental disorders, it seems plausible that left-handedness may frequently co-occur with DCD (Cairney et al., 2008). Therefore, the present study set out to identify whether an elevated frequency of left-handedness is a general trait of children with DCD when combining several small studies, to see if this relationship found in Cairney et al. (2008), Freitas et al. (2014), and Goez and Zelnik (2008) is robust across studies and different methodological approaches. The present study does not aim to test, or even discuss, the complete hypothesis of Geschwind and Behan (1982), as mentioned above, but instead tests one of the assumptions underlying that hypothesis: the elevated prevalence of left-handedness. Thus, it could be seen as a departure point for further testing given that an absence of such an elevated prevalence would cast considerable doubt upon the hypothesis.

## Materials and methods

The following methods and inclusion and exclusion criteria were adapted from Rysstad and Pedersen (2016), who conducted a meta-analysis on non-right handedness among individuals within ASD. Computerized searches were conducted in PubMed, PsycInfo and CINAHL databases with the purpose of identifying all relevant articles published in English, without imposing limits on the time interval.

The initial searches were traditional and description based. The search terms “Developmental coordination disorder” and “DCD” were each used in combination with the term “hand,^*^” indicating the prefix “hand” and any extension of the word. The searches identified a total of 170 studies in PubMed, 299 studies in PsycInfo and 117 studies in CINAHL, as can be seen in Table 1. In the initial searches, the range of publication years examined were between 1993 and 2018, being effectively limited by the inclusion criterion that articles should include children with DCD, a term that was introduced around 1990 and included in the DSM IV in 1994.

**Table 1:** Description based search and findings.

**Search strategy**	**Articles identified**	**Articles included**
1 “Developmental coordination” and hand^*^(limits humans, English written)		
*PubMed*	83	12
*PsycInfo*	161	17
*CINAHL*	68	8
2 “DCD” and hand^*^(limits humans, English written)		
*PubMed*	87	11
*PsycInfo*	138	12
*CINAHL*	49	6
Total	586	(66) 21^*^

* *Due to a considerable overlap in the results of the use of different search terms and overlap between the databases, as well as duplication of data between articles, the total number of unique articles included amounts to 21*.

Articles were considered relevant based on their titles and abstracts, as well as manual computerized searches in the full format articles with the key words “left,” “right,” “handedness,” “preference,” and “dominant.” The latter was done to secure that all studies reporting hand preference in DCD people without directly investigating the association would be identified.

Potentially relevant articles were obtained and assessed according to the following criteria:

Inclusion criteria:

1. Articles written in English
2. Empirical studies
3. Individuals with Developmental Coordination Disorder
4. Studies reporting distribution of left-handers versus right-handers in frequencies, or percent

Exclusion criteria:

1. Articles written in any other language than English
2. Reviews, books, theoretical papers, descriptive papers, theses
3. Diagnoses similar to DCD, including older terms, with somewhat similar but not identical diagnostic criteria.

Based on the results of the description-based searches, citation-based searches were conducted using Google Scholar. This strategy had previously proven to be much more effective in identifying relevant papers, compared with more traditional searches (see Rysstad and Pedersen, 2016 for details). Google Scholar includes articles from every other database and reports the citations for each article. All English written articles citing each of the already identified were examined using the same procedure as above in order to identify additional articles to include. The articles identified through the description-based searches had been cited by other articles a total of 889 times, as can be seen in Table 2. Twenty of these articles were ultimately included in the present dataset. Thus, to escape our description-based search and not be included, a paper would have to be published in a journal that is not indexed in any of the PsycInfo, PubMed or CINAHL databases, or not be identified by the chosen search terms. In order to escape our citation-based search, a paper would not have been cited by any of the papers that had already been identified as relevant from the description-based searches (which is definitely possible). However, neither would it have cited even one of the relevant papers (which would be highly unlikely).

**Table 2:** Citation based search and findings.

**Study**	**Citations**	**Articles included**
Adams et al., 2017	3	2
Armitage and Larkin, 1993	51	0
Asmussen et al., 2014	14	1
Cairney et al., 2008	28	0
Chang and Yu, 2010	56	3
Coats et al., 2015	3	0
Cox et al., 2015	7	0
Ferguson et al., 2014	15	2
Fuelscher et al., 2015	12	4
Goez and Zelnik, 2008	35	2
Hill, 1998	163	1
Hill and Bishop, 1998	54	1
Hodgson and Hudson, 2017	5	0
Hyde and Wilson, 2011a	44	8
Kashuk et al., 2017	0	0
Lust et al., 2006	38	2
Roche et al., 2011	9	0
Rodger et al., 2003	89	2
Rosenblum and Regev, 2013	14	0
Rosenblum et al., 2013	12	0
Smyth and Mason, 1997	99	5
van Swieten et al., 2010	74	2
Volman and Geuze, 1998	64	1
Total	889	(36) 20^*^

* *Unique papers, due to considerable overlap. Six articles identified through citation-based searches had already been identified and included in the description-based searches. In addition, a few papers were excluded due to duplication of data*.

Thus, the present dataset would be more than representative of papers on DCD, and it is difficult to imagine that papers not included would have a systematically different distribution of participants' handedness across groups compared with those included.

### Statistical approaches

Combined handedness distributions of left- and right-handers across the included studies were calculated as percentages and absolute numbers for the DCD (Table 3) and control groups (Table 4). The absolute numbers were compared by means of a chi-square test.

**Table 3:** Studies reporting distributions of handedness for individuals with Developmental Coordination Disorder.

**Study**	**Participants**	**Inclusion criteria**	**Measurement**	**Right-handed**	**Left-handed**
Adams et al., 2016	*N =* 30 (20 male, 10 female); age *=* 6–10 years	*DSM-V* criteria, MABC-2 ≤ 5th centile	Writing hand, Procedure described in the MABC-2	93.3% (28)	6.7% (2)
Armitage and Larkin, 1993^*^	*N =* 27; age *=* 5–9	Referred to or involved in movement program	Drawing, erasing, throwing a ball, and dealing cards (Porac and Coren, 1981)	92.6% (25)	7.4% (2)
Asmussen et al., 2014	*N =* 10 (10 male); age *=* 9–12 years	*DSM-IV* criteria DSDQ MABC	Observed hand to write name and hand used to catch a ball	70.0% (7)	30.0% (3)
Bonney et al., 2017	*N =* 57 (29 male, 28 female); age *=* 6–10 years	DSM-V criteria	Not explicitly stated	93.0% (53)	7.0% (4)
Cairney et al., 2008	*N =* 19; age *=* 11years	MABC BOTMP-SF test ≤ 5th centile Kaufman Brief Intelligence Test	Observed hand preference throughout the testing process	63.2% (12)	36.8% (7)
Chang and Yu, 2010	*N =* 33 (18 male, 15 female); age *=* 6–8 years	MABC, DCDQ	Not explicitly stated	87.9% (29)	12.1% (4)
Coats et al., 2015	*N =* 10 (6 male, 4 female); age *=* 7–10 years	*DSM-IV* criteria	Writing hand	90.0% (9)	10.0% (1)
Cox et al., 2015	*N =* 20 (15 male, 5 female); age *=* 6–12 years	*DSM-V* criteria MABC-2 ≤ 15 th centile	The Edinburgh Handedness Inventory (Oldfield, 1971)	85.0% (17)	15.0% (3)
de Oliveira et al., 2014	*N =* 11(6 male, 5 female); age *=* 16–26 years	Previously diagnosed with DCD, MABC-2	Not explicitly stated	81.8% (9)	18.2% (2)
Debrabant et al., 2013	*N =* 17 (14 male, 3 female); age *=* 7–10 years	MABC-2 ≤ 5th centile The Wechsler Intelligence Scale for children	Not explicitly stated	82.4% (14)	17.6% (3)
Engel-Yeger and Hanna Kasis, 2010	*N =* 37 (26 male, 11 female); age *=* 5–9 years	Diagnose by pediatrician/developmental neurologist *DSM-IV* criteria MABC ≤ 15 th centile	Demographic questionnaire, not explicitly stated who completed these	94.6% (35)	5.4% (2)
Ferguson et al., 2014	*N =* 70 (36 male, 34 female); age *=* 6–10 years	Problematic daily life motor function according to teacher and/or parent MABC-2 ≤ 5th centile	Not explicitly stated	91.4% (64)	8.6% (6)
Ferguson et al., 2015	*N =* 30 (16 male, 14 female); age *=* 6–10 years	*DSM-IV* criteria MABC-2 ≤ 5th centile	The hand normally used for writing or drawing	93.3% (28)	6.7% (2)
Fuelscher et al., 2015	*N =* 17 (8 male, 9 female); age *=* 8–12	*DSM-V* criteria McCarron Assessment of Neuromuscular Development ≤ 15th centile	Not explicitly stated	88.2% (15)	11.8% (2)
Goez and Zelnik, 2008^*^	*N =* 85 age *=* 5–17 years	*DSM-IV* criteria	Examinations of the preferred writing hand, preferred hand for throwing a ball, and preferred hand for holding a table spoon	64.7% (55)	35,3% (30)
Hill, 1998	*N =* 11 (8 male, 3 female); age *=* 5–13 years	Diagnosed with DCD	Not explicitly stated	72.7% (8)	27.3% (3)
Hill and Bishop, 1998	*N =* 12 (9 male, 3 female); age *=* 7–11 years	M-ABC ≤ 15th centile Raven's Progressive Matrices ≥ 80 CELF-R Repeating Sentences ≥ 80	Writing hand, Handedness questionnaire based on the Edinburgh Handedness Inventory (Oldfield, 1971), completed by the children's parents	83.3% (10)	16,7% (2)
Hodgson and Hudson, 2017	*N =* 12 (4 male, 8 female); age *=* 18–43 years	Self-report on the Adult Developmental Coordination Disorder Checklist	21-item handedness questionnaire (Flowers and Hudson, 2013)	75.0% (9)	25.0% (3)
Hyde and Wilson, 2011a	*N =* 17 (8 male, 9 female); age *=* 7–12 years	*DSM-IV* criteria McCarron Assessment of Neuromuscular Development ≤ 10th centile	Not explicitly stated (but see Hyde and Wilson, 2011b, below)	76.5% (13)	23.5% (4)
Hyde and Wilson, 2011b	*N =* 13 (4 male, 9 female); age *=* 8–12 years	*DSM-IV* criteria McCarron Assessment of Neuromuscular Development ≤ 10th centile	McCarron Assessment of Neuromuscular Development	69.2% (9)	30.8% (4)
Hyde and Wilson, 2013	*N =* 18 (7 male, 11 female); age *=* 8–12 years	*DSM-IV criteria* McCarron Assessment of Neuromuscular Development ≤ 10^th^ centile	Reaching hand	77.8% (14)	22.2% (4)
Kashuk et al., 2017	*N =* 12 (5 male, 7 female); age *=* 18–40 years	McCarron Assessment of Neuromuscular Development Adult Dyspraxia/Developmental Coordination Disorder Checklist	Not explicitly stated	75.0% (9)	25.0% (3)
Lust et al., 2006^*^	*N =* 7; age *=* 9–11 years	M-ABC ≤ 15th centile	Handedness Inventory (Van Strien, 1992)	85.7% (6)	14.3% (1)
Maleki and Zarei, 2016	*N =* 53 (32 male, 21 female); age *=* 7–11 years	Persian version of motor observation questionnaire for teachers Diagnosis by psychiatrists	Not explicitly stated	83.0% (44)	17.0% (9)
Roche et al., 2011	*N =* 10 (7 male, 3 female); age *=* 6–8 years	Diagnosed with DCD MABC ≤ 15th centile Woodcock-Johnson Psycho-Educational Battery	Annett Handedness Questionnaire (1970)	90.0% (9)	10% (1)
Rodger et al., 2003	*N =* 20 (12 male, 8 female); age *=* 4–8 years	MABC ≤ 15th centile	Observed hand preference during writing tasks	70.0% (14)	30.0% (6)
Rosenblum and Regev, 2013	*N =* 21 (13 male, 8 female); age *=* 7–10 years	Educators' or clinicians' reports based on *DSM-IV* criteria MABC ≤ 15th centile	Not explicitly stated (but see Rosenblum et al., 2013, 2017, below)	95.2% (20)	4.8% (1)
Rosenblum et al., 2013	*N =* 29 (24 male, 5 female); age *=* 11–12 years	*DSM-IV* criteria MABC ≤ 5th centile	Demographic questionnaire completed by the children's parents	96.6% (28)	3.4% (1)
Rosenblum et al., 2017^*^	*N =* 27; age *=* 4–6 years	Diagnosed according to *DSM-V* criteria and MABC ≤ 5th centile	Demographic questionnaire completed by the children's parents	92.6% (25)	7.4% (2)
Ruddock et al., 2016	*N =* 62; age *=* 6–12 years	McCarron Assessment of Neuromuscular Development ≤ 15th centile	Manual dexterity items of McCarron Assessment of Neuromuscular Development Hand used during writing	91.9% (57)	8.1% (5)
Schoemaker et al., 2001	*N =* 19 (11 male, 8 female); age *=* 6–11 years	MABC ≤ 15th centile and ≤ 5th centile	Not explicitly stated	73.7% (14)	26.3% (5)
Sinani et al., 2011	*N =* 45 (29 male, 16 female); age *=* 9–11 years	*DSM-IV-TR* criteria MABC ≤ 15 th centile	Hand used for holding a pen	88.9% (40)	11.1% (5)
Smits-Engelsman et al., 2016	*N =* 17 (9 male, 8 female) age *=* 6–10 years	*DSM-V* criteria MABC ≤ 5th centile	Not explicitly stated, (but see same authors in Ferguson et al., 2015, above)	94.1% (16)	5.9% (1)
Smyth and Mason, 1997	*N =* 96 (59 male, 37 female); age *=* 4–8 years	MABC ≤ 5th centile The British Ability Scale	Not explicitly stated	89.6% (86)	10.4% (10)
van Swieten et al., 2010	*N =* 27 (20 male, 7 female); age *=* 6–13 years	*DSM-IV* criteria MABC ≤ 5th centile	Writing hand	88.9% (24)	11.1% (3)
Van Waelvelde et al., 2006	*N =* 36 (22 male, 14 female); age *=* 9–10 years	MABC ≤ 5th centile	Indicated by the children	86.1% (31)	13.9% (5)
Volman and Geuze, 1998	*N =* 24 (21 male, 3 female); age *=* 7–12 years	MABC ≤ 15th centile	Hand used for writing	79.2% (19)	20.8% (5)
Whitall et al., 2008	*N =* 10 (7 male, 3 female); age *=* 6–7 years	Diagnosed with DCD MABC ≤ 15th centile DCDQ	Not explicitly stated	90.0% (9)	10.0% (1)
Total	*N =* 1071			85.3% (914)	14.7% (157)

* *The study included mixed-handers, who were omitted from the current study. N = sample excluding mixed-handers*.

**Table 4:** Distribution of handedness in control groups.

**Study**	**Participants of control group**	**Measurement**	**Right-handed**	**Left-handed**
Adams et al., 2016	*N =* 90 (50 male, 40 female)	Matched by age	96.7% (87)	3.3% (3)
Armitage and Larkin, 1993^*^	*N =* 31 age 5–9 years	Grouped according to coordination and age	93.5% (29)	6.5% (2)
Asmussen et al., 2014	*N =* 9 (9 male)	Typically developing children from the school system	88.9% (8)	11.1% (1)
Bonney et al., 2017	*N =* 54 (28 male, 26 female)	Typically developing children from the same school as the DCD children	96.3% (52)	3.7% (2)
Cairney et al., 2008^**^				
Chang and Yu, 2010	*N =* 22 (12 male, 10 female)	Matched by age and sex	86.4% (19)	13.6% (3)
Coats et al., 2015	*N =* 10 (5 male, 5 female)	Matched by age	90.0% (9)	10.0% (1)
Cox et al., 2015	*N =* 16 (6 male, 10 female)	Typically developing children, MABC score ≥ 15 th centile	87.5% (14)	12.5% (2)
de Oliveira et al., 2014	*N =* 11 (6 male, 5 female)	Matched by sex and similar age	100.0% (11)	0.0% (0)
Debrabant et al., 2013	*N =* 17 (14 male, 3 female)	Matched by age and sex	88.2% (15)	11.8% (2)
Engel-Yeger and Hanna Kasis, 2010	*N =* 37 (26 male, 11 female)	Matched by sex, age and socio-economic status	91.9% (34)	8.1% (3)
Ferguson et al., 2014	*N =* 70 (35 male, 35 female)	Matched by age and sex	92.9% (65)	7.1% (5)
Ferguson et al., 2015	*N =* 30 (15 male, 15 female)	Matched by age and sex	93.3% (28)	6.7% (2)
Fuelscher et al., 2015	*N =* 17 (8 male, 9 female)	Matched by age	88.2% (15)	11.8% (2)
Goez and Zelnik, 2008^**^				
Hill, 1998	*N =* 25 (14 male, 11 female)	Matched by age, sex, non-verbal IQ, and language ability	88.0% (22)	12.0% (3)
Hill and Bishop, 1998	*N =* 26 (15 male, 11 female)	Matched by age, sex ratio, and non-verbal ability	80.8% (21)	19.2% (5)
Hodgson and Hudson, 2017	*N =* 12 (5 male,7 female)	General student and staff population	91.7% (11)	8.3% (1)
Hyde and Wilson, 2011a	*N =* 27 (14 male, 13 female)	Matched by age	96.3% (26)	3.7% (1)
Hyde and Wilson, 2011b	*N =* 13 (7 male, 6 female)	Matched by age	100.0% (13)	0.0% (0)
Hyde and Wilson, 2013	*N =* 18 (8 male, 10 female)	Matched by age	94.4% (17)	5.6% (1)
Kashuk et al., 2017	*N =* 11 (6 male, 5 female)	General student and staff population	72.7% (8)	27.3% (3)
Lust et al., 2006^*^	*N =* 5	Matched by age and sex	100% (5)	0.0% (0)
Maleki and Zarei, 2016^**^				
Roche et al., 2011	*N =* 10 (7 male, 3 female)	Matched by age and sex	90.0% (9)	10.0% (1)
Rodger et al., 2003^**^				
Rosenblum and Regev, 2013	*N =* 21 (13 male, 8 female)	Matched by age, sex, and school	95.2% (20)	4.8% (1)
Rosenblum et al., 2013	*N =* 29 (24 male, 5 female)	Matched by age, sex, and school	93.1% (27)	6.9% (2)
Rosenblum et al., 2017^*^	*N =* 33	Matched by age, sex and socio-economic status	90.9% (30)	9.1% (3)
Ruddock et al., 2016	*N =* 109 (48 male, 61 female)		94.5% (103)	5.5% (6)
Schoemaker et al., 2001	*N =* 19 (11 male, 8 female)	Matched by age and sex	84.2% (16)	15.8 (3)
Sinani et al., 2011	*N =* 24 (16 male, 8 female)	Matched by age, sex, ethnicity and academic ability	87.5% (21)	12.5% (3)
Smits-Engelsman et al., 2016	*N =* 18 (9 male, 9 female)	Matched by age	94.4% (17)	5.6% (1)
Smyth and Mason, 1997	*N =* 91 (54 male, 37 female)	Matched by age, sex, and performance on the The British Ability Scale	91.2% (83)	8.8% (8)
van Swieten et al., 2010	*N =* 70 (35 male, 35 female)	Typically developing children	87.1% (61)	12.9% (9)
Van Waelvelde et al., 2006	*N =* 36 (22 male, 14 female)	Matched by age and sex	97.2% (35)	2.8% (1)
Volman and Geuze, 1998	*N =* 24 (20 male, 4 female)	Individually matched	83.3% (20)	16.7% (4)
Whitall et al., 2008	*N =* 10 (7 male, 3 female)	Matched by age and sex	90.0% (9)	10.0% (1)
Total	*N =* 1,045		91.9% (960)	8.1% (85)

* *The study included mixed-handers, who were omitted from the current study. N = sample excluding mixed-handers*.

** *No control group/not included in the analysis*.

## Results

Thirty-eight studies were included in the present review, containing handedness distributions across 1071 persons with DCD, as presented in Table 3. The earliest of the studies was published in 1993, and the most recent were published in 2017. The ages of the participants varied between 4 and 43 years, but the vast majority of the studies included children between 7 and 12 years. Three studies included adults. On closer inspection, it was detected that four papers by the same group of authors included the same participants, or samples of participants that had almost complete overlap. Thus, only one of the studies (Adams et al., 2016) was included in the present dataset.

Only four studies reported mixed-handedness. Participants with mixed-handedness were omitted from the analysis. This was the case for a total of 44 mixed-handers (2.0% of the total sample). Thirty-two belonged to the DCD-groups and 12 were controls. The studies defined mixed-handedness differently. Regardless of the categorization used in a study, however, DCD-individuals and controls were categorized in the same manner, so the relative distribution of left- and right-handers across these groups would not seem to be affected.

The controls of the studies including such groups were used for comparison, as can be seen in Table 4. Four studies did not provide data on such, namely Cairney et al. (2008), Goez and Zelnik (2008), Maleki and Zarei (2016), and Rodger et al. (2003). Several studies also included more than one control group (e.g., younger controls, adults or both). In those cases, only one—preferably the one matched by age and sex and other possible variables with the study participants—were included for the purpose of the present study, as can be found in Table 4. Across 24 studies, 1,045 control participants were included.

A male-female ratio in favor of the male distribution was evident in both the DCD group and control group. However, specific gender analyses were not possible because most of the studies lacked reports of the gender distribution across handedness-categories.

Among 1,071 participants with DCD, 14.7% were classified as left-handers, compared to 8.1% among 1045 participants in the control groups. The handedness distribution varied some: the highest reported number of left-handers in a DCD-group was found in Cairney et al. (2008), with 36.8%, while Rosenblum et al. (2013) identified only 3.4% left-handers. The distribution of DCD participants across handedness-categories proved to be significantly different from that of the control group, χ^2^ = 22.2345, *p* = 0.000002.

## Discussion

An overrepresentation of left-handers in DCD children has repeatedly been assumed. However, due to primarily small individual samples reporting handedness within this population, no other study has been large enough to conclude that there is actually an elevated prevalence of left-handers in this group, and no one has yet combined the results in a meta-analysis or review. Sample sizes varied across the studies, with the smallest sample size found in Lust et al. (2006) (*N* = 7) and the largest in Smyth and Mason (1997) (*N* = 96). Twenty-eight out of the 38 studies had sample sizes of 30 participants or below, with the vast majority including fewer than 20 participants. The results also appear to be robust across studies and do not seem to be related to differences with respect to measures of handedness, categorizations of participants for inclusion in the DCD-group, or participants' age or sex. Therefore, it should be possible to conclude that an elevated frequency of left-handedness is a general trait of individuals within DCD.

Participants' handedness was classified as right-handed or left-handed based on a variety of measures across studies, such as writing or drawing hand, The Edinburgh Handedness Inventory (Oldfield, 1971), the Movement ABC (Henderson and Barnett, 1992), the Annett Handedness Questionnaire (Annett, 1970), Porac and Coren's (1981) questionnaire or observed hand throughout the testing process. There is, seemingly, no trend related to classification of handedness, as the handedness distributions varied across samples even when using the same measure of handedness, indicating that differences across measures are not systematic.

Diagnostic criteria for DCD varied some across the included studies, with the vast majority using DSM-IV criteria and/or a version of the MABC. The cut-off value of the MABC varied somewhat between ≤ 16th (Cairney et al., 2008) and 5th centile (e.g., Debrabant et al., 2013). Even when using the same criteria for DCD, handedness distributions varied across samples without there, seemingly, being any trend related to inclusion criteria of the respective samples.

Most participants were between the ages of 4 and 12 years old (see Table 3 for details) and there is no evidence for the effects of age upon the distributions across handedness categories. Also, most studies included control participants who were age matched with the DCD-groups.

More boys than girls (2:1) are diagnosed with DCD (Barnhart et al., 2003) and there are also more left-handed boys than girls, with an estimate of 1.23 for the ratio of male to female left-to right-handedness odds (Papadatou-Pastou et al., 2008). This could lead one to believe that the difference between the groups would be due to the sex ratio. However, although the samples' skewed sex ratios, in part, could explain why there are more left-handers in the DCD-group, the differences in handedness distributions are still much larger than what might be expected based on sex differences alone. Furthermore, and even more importantly, the majority of the studies matched their controls by both sex and age; thus, there were equally as many boys among the controls. Moreover, there seems to be no clear trend across the studies that handedness distribution is dependent on the sex ratio.

The prevalence of left-handedness within the DCD-group was somewhat smaller than the prevalence found for ASD in Rysstad and Pedersen (2016), with 16% pure left-handers. However, four of the studies in the current analysis included participants reporting mixed- or ambidextrous handedness. Although mixed- and ambidextrous handedness was not included in the data material, these studies indicate that non-right-handedness may be an even more prominent trait of DCD children than left-handedness. In Goez and Zelnik (2008), 13% of the children were classified as ambidextrous, giving a total of 44% non-right-handers. Armitage and Larkin (1993) reported 30% mixed-handers in 5- to 6-year-olds and 35% in 8- to 9-year-olds, giving a total of 40% non-right-handedness in the younger group, while Lust et al. (2006) also reported 30% ambidextrous handedness, totalling 40% non-right-handedness.

Thus, DCD has a slightly lower proportion of left-handers than found in ASD, and there are also indications of less mixed- and ambidextrous handedness in DCD than in ASD (Rysstad and Pedersen, 2016). However, there is a higher proportion of left-handers in the DCD group than was found in dyslexics (Eglinton and Annett, 1994), as well as indications of a much larger proportion of mixed- and ambidextrous handedness compared with this group. Although a higher proportion of left-handers in these groups have been found in comparison with healthy controls, there is a markedly higher rate of mixed- and ambidextrous handedness, which indicates that inconsistent handedness may be a bigger problem than consistent left-handedness in these groups, as was argued by Prichard et al. (2013).

In the current meta-analysis, we have established there is an elevated frequency of left-handers in DCD, thus supporting the theory of Geschwind and Behan (1982). However, the results say nothing about the direction of the association between the variables. One could, for example, speculate that some diagnostic criteria for DCD favor right-handers, thereby giving left-handers an elevated risk of being diagnosed with DCD. The inclusion of matched control groups (with a few exceptions) that have undergone the same testing procedures as the DCD-groups makes this rather unlikely, as well as the fact that the findings seem robust across different inclusion criteria.

The fact that handedness distributions of DCD children are compared with the corresponding distributions within the groups of controls included in each study is, in fact, a major strength of the present study. This secures that whatever differences found across studies with respect to measures, handedness categorization, sex, age, and other possible confounders would be accounted for, and thus should not affect the present results. Another major strength is that the included studies from which handedness distributions were extracted did not generally have the scope to study handedness in DCD-children *per se*. Rather, they studied a host of other more or less related topics, and reported handedness (with only a very few exceptions) as a background variable. Hence, the handedness distributions reported in each individual paper would appear relatively unbiased with respect to the bigger picture of a general elevated prevalence of left-handedness.

In summary, the current study gives support to a notion that there is a link between left-handedness and several developmental disorders, including DCD. The data, however, cannot further the discussions about a shared underlying mechanism, as proposed by Geschwind and Behan (1982).

## Conclusion

The distribution of DCD participants across handedness-categories was significantly different from that of the control groups. As discussed, the number of participants in each individual study was too small for any generalizations to be made, but the combined number of participants gives the largest sample addressing the association between handedness and DCD currently available. Results appear robust across inclusion criteria for DCD and measures of handedness, as well as age and sex. This suggests that an elevated prevalence of left-handedness in individuals with DCD has been proved valid in the current study and gives support for an assumption of a shared underlying mechanism of disorders in some clinical groups. The prevalence of left-handedness within the DCD-group is lower than that reported for ASD, and larger than in dyslexia. However, no data in the present study can support or contradict the proposed assumption of a common cause of the various disorders. The present results could, however, constitute a starting point for testing the hypothesis of such a common factor, as one of the requirements would be an elevated prevalence of left-handedness, and without such the hypothesis could more or less be rejected.

## Author contributions

MD, AP, and HL contributed to the conception and design of the study. MD carried out the searches, but conferred and discussed with AP throughout the process. MD, AP, and HL analyzed and/or interpreted the data. MD wrote the first draft of the manuscript and AP wrote sections of the manuscript. All authors contributed to manuscript revision and read and approved the submitted version.

### Conflict of interest statement

The authors declare that the research was conducted in the absence of any commercial or financial relationships that could be construed as a potential conflict of interest.
